# C57Bl/6N mice have an attenuated lung inflammatory response to dsRNA compared to C57Bl/6J and BALB/c mice

**DOI:** 10.1186/s12950-023-00331-4

**Published:** 2023-02-21

**Authors:** Sofia Malm Tillgren, Juan José Nieto-Fontarigo, Samuel Cerps, Sangeetha Ramu, Mandy Menzel, Irma Mahmutovic Persson, Anja Meissner, Hamid Akbarshahi, Lena Uller

**Affiliations:** 1 Department of Experimental Medical Science, Unit of Respiratory immunopharmacology, Lund University, Lund, Sweden; 2 Wallenberg Center for Molecular Medicine, Lund University, Lund, Sweden; 3 Department of Physiology, Institute of Theoretical Medicine, Medical Faculty, University of Augsburg, Augsburg, Germany; 4 Department of Experimental Medical Science, Lund University, Lund, Sweden; 5 Department of Clinical Sciences, Division of Respiratory Medicine and Allergology, Lund University, Lund, Sweden

**Keywords:** BALB/c, C57Bl/6J, C57Bl/6N, dsRNA, Poly(I:C), Inflammation, Lung, Mouse models, Respiratory viral infection

## Abstract

**Background:**

Lower respiratory infections caused by ssRNA viruses are a major health burden globally. Translational mouse models are a valuable tool for medical research, including research on respiratory viral infections. In in vivo mouse models, synthetic dsRNA can be used as a surrogate for ssRNA virus replication. However, studies investigating how genetic background of mice impacts the murine lung inflammatory response to dsRNA is lacking. Hence, we have compared lung immunological responses of BALB/c, C57Bl/6N and C57Bl/6J mice to synthetic dsRNA.

**Methods:**

dsRNA was administered intranasally to BALB/c, C57Bl/6N and C57Bl/6J mice once/day for three consecutive days. Lactate dehydrogenase (LDH) activity, inflammatory cells, and total protein concentration were analyzed in bronchoalveolar lavage fluid (BALF). Pattern recognition receptors levels (TLR3, MDA5 and RIG-I) were measured in lung homogenates using RT-qPCR and western blot. Gene expression of IFN-β, TNF-α, IL-1β and CXCL1 was assessed in lung homogenates by RT-qPCR. ELISA was used to analyze protein concentrations of CXCL1 and IL-1β in BALF and lung homogenates.

**Results:**

BALB/c and C57Bl/6J mice showed infiltration of neutrophils to the lung, and an increase in total protein concentration and LDH activity in response to dsRNA administration. Only modest increases in these parameters were observed for C57Bl/6N mice. Similarly, dsRNA administration evoked an upregulation of MDA5 and RIG-I gene and protein expression in BALB/c and C57Bl/6J, but not C57Bl/6N, mice. Further, dsRNA provoked an increase in gene expression of TNF-α in BALB/c and C57Bl/6J mice, IL-1β only in C57Bl/6N mice and CXCL1 exclusively in BALB/c mice. BALF levels of CXCL1 and IL-1β were increased in BALB/c and C57Bl/6J mice in response to dsRNA, whereas the response of C57Bl/6N was blunt. Overall, inter-strain comparisons of the lung reactivity to dsRNA revealed that BALB/c, followed by C57Bl/6J, had the most pronounced respiratory inflammatory responses, while the responses of C57Bl/6N mice were attenuated.

**Conclusions:**

We report clear differences of the lung innate inflammatory response to dsRNA between BALB/c, C57Bl/6J and C57Bl/6N mice. Of particular note, the highlighted differences in the inflammatory response of C57Bl/6J and C57Bl/6N substrains underscore the value of strain selection in mouse models of respiratory viral infections.

**Supplementary Information:**

The online version contains supplementary material available at 10.1186/s12950-023-00331-4.

## Background

Human viral respiratory infections and lower respiratory tract infections (LRTI) are a leading cause of mortality and morbidity globally [1]. LRTIs are most commonly caused by single stranded-RNA (ssRNA) viruses including respiratory syncytial virus (RSV), parainfluenza, rhinovirus (RV) and coronavirus [2]. Respiratory infections are also the most predominant triggers of asthma and chronic obstructive pulmonary disease (COPD) exacerbations, accounting for the majority of acute hospitalizations among these patients [3, 4].

As ssRNA viruses replicate inside host cells, double stranded RNA (dsRNA) is formed as an intermediate molecule [5]. dsRNA will then be recognized by pattern recognition receptors (PRRs) including endosomal toll-like receptor 3 (TLR3) and cytosolic receptors melanoma differentiation-associated protein 5 (MDA5) and retinoic acid-inducible gene I (RIG-I), which will initiate and orchestrate innate anti-viral and inflammatory responses [6]. Poly(I:C) is a synthetic dsRNA molecule widely used to mimic the innate inflammatory responses to ssRNA virus. Poly(I:C) has previously been shown to induce inflammation and impair lung function in mice, and serves as a suitable surrogate for ssRNA viral infection [7, 8]. Mouse models using poly(I:C) is therefore valuable when studying early innate responses orchestrated by TLRs.

Mouse models of respiratory viral infections have contributed extensively to knowledge on the pathogenesis and identification of host factors that are crucial for protection against severe viral-induced disease [9–11]. The inbred BALB/c mouse strain is well used to study viral infections, and have contributed to knowledge on the inflammatory response to different RV serotypes, as well as SARS-CoV-2 pathogenesis and vaccine development [9, 12]. Similarly, the C57Bl/6 mouse strain is also frequently used and have been used to investigate inflammatory responses to RSV, as well as mechanisms of RV-induced asthma exacerbations [10, 13]. Previous studies have highlighted that BALB/c and C57Bl/6 strains have separate susceptibility and inflammatory responses to some respiratory viruses. Yasui et al. have demonstrated different cytokine expression patterns of BALB/c and C57Bl/6J mice towards SARS-CoV-2 infection, and Watkiss et al. have reported different susceptibilities to pneumonia virus of mice (PVM) [14, 15].

The C57Bl/6 strain was the first strain to have its genome sequenced and is one of the most frequently used strains in medical research. Although the substrains of C57Bl/6 are genetically similar, differences have been identified, including 34 SNPs and 2 indels that separate the coding sequences of the C57Bl/6N and C57Bl/6J strains [16]. Among these, the C57Bl/6J strain have an in-frame five-exon deletion of the nicotinamide nucleotide transhydrogenase (nnt), and loss of this protein has been attributed to an increase in inflammation [17]. As a consequence, comparing results from different substrains of C57Bl/6 can be misleading. Unfortunately, inadequate reporting of substrain identity is common. Hence, discrepancies in published results from similar experiments can be found, a possible consequence of the use of different substrains [18, 19].

Reports comparing the immune responses to respiratory viral infections of C57Bl/6N and C57Bl/6J in a single study are lacking. Comparisons of the inflammatory signature of the brain in response to intraperitoneal administration of dsRNA have been done by Warden et al., where they report distinct differences in the immune signature of these two strains [20]. Moreover, Eisfeld et al. have compared the susceptibility and pathology of influenza infection in C57Bl/6J and C57Bl/6NJ mice [21]. The C57Bl/6NJ is however a substrain of the C57Bl/6N, and hence, these strains also differ genetically. Furthermore, in some studies of BALB/c and C57Bl/6 comparisons, proper substrain specification is missing, as in the study by Watkiss et al. referenced above [15]. The aim of this study was therefore to compare the pulmonary innate inflammatory responses of C57Bl/6J, C57Bl/6N and BALB/c, three of the most frequently used mouse strains, in one single study.

We chose to use the viral mimic poly(I:C) to enable a wider applicability and to feature early innate responses. By doing this, we aimed to generate a study that can be used as an aid in strain selection for mouse studies involving respiratory ssRNA virus. By highlighting the differences and similarities in the immunological response, we believe that our study can improve consistency of experimental data, and improve translational discovery research.

## Methods and materials

### Animals

8–10-week-old female C57Bl/6N (Janvier Labs, Le-Genest-Saint-Isle, France), C57Bl/6J (Charles River Laboratories, Sulzfeld, Germany) and BALB/c (Taconic Biosciences, Ejby, Denmark) mice were used in this study. Mice were acclimatized for 1 week prior experiments. Mice were housed and experiments were performed according to the European Parliament and Council Directive 2010/63/EU, the Swedish Animal Welfare Act (Djurskyddslag 1988:534), the Swedish Animal Welfare Ordinance (Djurskyddsförordning 1988:539) and Institutional Animal Care and Use Committee (IACUC) guidelines, and was approved by the Malmö/Lund Animal Experimental Ethics Committee at the Lund District Court in Sweden (approval number M 6436/2017). The mice were housed in groups of 4–5, with a 12-h light and dark cycle and had free access to food and water at the animal facility at Lund University.

**Fig. 1:**
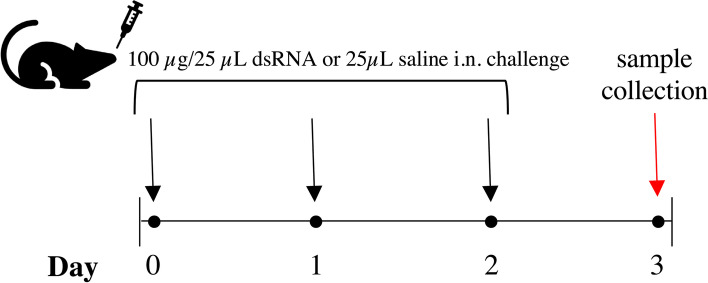
Study design for intranasal (i.n.) dsRNA or saline administration to mice. dsRNA (100 µg) or saline were i.n. administered to C57Bl/6J, C57Bl/6N or BALB/c mice once per day for three consecutive days. At day 3, mice were euthanized following BALF collection and lung tissue dissection for further analysis

Hundred micrograms (100 µg) polyinosine-polycytidylic acid (poly(I:C); InVivogen, San Diego, USA) dissolved in 25 µL saline, or 25 µL saline (as vehicle control) were administered intranasally (i.n.) for three consecutive days as previously described [22, 23]. Twenty-four hours after last administration mice were euthanized by intraperitoneal injection of 100 µL of 60 mg/ml pentobarbital (Apoteket AB, Stockholm, Sweden) (Fig. 1).

In total, 30 mice were divided into 6 different groups: C57Bl/6N:saline (*n* = 5), C57Bl/6N:dsRNA (*n* = 5), C57Bl/6J:saline (*n* = 5), C57Bl/6J:dsRNA (*n* = 5), BALB/c:saline (*n* = 5) and BALB/c:dsRNA (*n* = 5).

### Bronchoalveolar lavage fluid and lung dissection

Bronchoalveolar lavage fluid (BALF) was collected by rinsing lungs with PBS. Following BALF collection, right lung lobes were dissected and snap frozen in liquid nitrogen followed by storage in -80 °C. Firstly, a mechanical homogenization of the lung was performed using an OmniPrep Rotor Stator Generator (Omni International, Waterbury, USA). For ELISA, samples were homogenized in PBS with a protease inhibitor cocktail (1 tablet/20 mL PBS) (Roche Diagnostics GmbH, Mannheim, Germany). Samples for western blot were homogenized in lysis buffer (1% TritonX-100, 10 mM Tris–HCl, 50 mM NaCl, 5 mM EDTA, 30 mM sodium pyrophosphate, 50 mM NaF, 0.1 mM Na_3_VO_4_) with 1% protease and phosphatase inhibitors (Sigma-Aldrich, Stockholm, Sweden). RT-qPCR samples were homogenized in lysis buffer (Macherey–Nagel, Düren, Germany) with 1% β-mercaptoethanol (Sigma-Aldrich, Stockholm, Sweden).

### Differential cell count in BALF

BALF was centrifuged at 230 × g 10 min at 4 °C, supernatants were collected and cell pellets were resuspended in PBS. Cell counts were determined using a NucleoCounter (Chemometec, Allerod, Denmark) and 50,000 cells were centrifuged (6 min at 450 × g) onto microscope slides using cytospin funnels and a Shandon Cytospin 3 (Marshall Scientific, Hampton, USA). Cytospin slides were stained with Kwik-Diff stain (Epredia, Kalamazoo, USA) and differential cell count of 400 cells was determined under a light microscope (Olympus CKX53, LRI Olympus, Lund, Sweden).

### RNA extraction and gene expression analysis

RNA from homogenized lungs was extracted using the Nucleospin® RNA II kit (Macherey–Nagel, Düren, Germany), and 1 µg total RNA was reversely transcribed using the High-capacity cDNA reverse transcription kit (Applied Biosystems by Thermo Fisher Scientific Baltics UAB, Vilnius, Lithuania). Gene expression analysis was performed by RT-qPCR on an AriaMx qPCR system (Stratagen, La Jolla, USA) with standard cycling parameters. Primers were obtained from Qiagen (Stockholm, Sweden) and PrimerDesign (Southampton, United Kingdom), a full list can be found in Table 1. ΔCt for each gene was calculated by normalization to the house-keeping gene β-actin and ΔΔCt was calculated by normalization to the mean ΔCt of each control group (saline).

**Table 1:** Primer sequence list

Gene	Vendor	Sequence
TNF-α	PrimerDesign	AGCCAGGAGGGAGAACAGA (forward) CAGTGAGTGAAAGGGACAGAAC (reverse)
RIG-I	PrimerDesign	CGATATTTTGAAAGACTTGGGTACA (forward) ATGGCTCCGTTGTTGAGATTG (reverse)
TLR3	PrimerDesign	AAGTTATTCGCCCTCCTCTTGA (forward) AGATTCTGGATGCTTGTGTTTGA (reverse)
IFN-β	PrimerDesign	ATGGAAAGATCAACCTCACCTAC (forward) GGATGGCAAAGGCAGTGTAA (reverse)
IFN-λ	PrimerDesign	ACCAGGCTCCCAGTGGAA (forward) and TTTTTGAAGGCCTGCAGCTCT (reverse)
IL-1β	Qiagen	Mm_Il1b_2_SG QuantiTect Primer Assay (QT01048355)
MDA5	Qiagen	Mm_Ifih1_1_SG QuantiTect Primer Assay (QT00156338)
β-actin	Qiagen	Mm_Actb_1_SG QuantiTect Primer Assay (QT00095242)
CXCL1	Qiagen	Mm_Cxcl1_1_SG QuantiTect Primer Assay (QT00115647)
Viperin	Qiagen	Mm_Rsad2_1_SG QuantiTect Primer Assay (QT00109431)

Table 1. List indicating primer sequence (where available) and accession number for primers used in this work

### Total protein and Enzyme-Linked Immunosorbent Assays (ELISA) analysis

Total protein concentration was determined in BALF supernatants and lung homogenates using Pierce BCA assay (Thermo Scientific, Waltham, USA). Concentrations of CXCL1 and IL-1β were measured using ELISA with kits from R&D systems (Abingon, UK), according to manufacturer’s instructions.

### Lactate dehydrogenase activity assay

A cytotoxicity Detection kit (Roche, Stockholm, Sweden) was used to measure lactate dehydrogenase (LDH) activity in BALF, according to manufacturer’s instructions.

### Western blot analysis

Lung homogenates were analyzed for protein expression of PRRs by western blot. Equal amounts of protein were boiled at 95° (5 min) in laemmli buffer (Sigma Aldrich, Stockholm, Sweden), and loaded onto Mini-protean TGX stain free, 4–20% gels (Bio-rad, Stockholm, Sweden) and separated by gel electrophoresis using a Mini protean tetra cell electrophoresis chamber (Bio-Rad). Proteins were transferred to Trans-Blot turbo (Bio-Rad) membranes using the Trans-Blot turbo system (Bio-Rad). Membranes were cut at 50 kDa according to the Spectra Broad Range protein ladder (Thermo Scientific, Waltham, USA) and blocked in EveryBlot blocking buffer (Bio-Rad) for 10 min. Membranes were then incubated at 4 °C over-night with primary anti-mouse rabbit antibodies from Cell Signaling (Danvers, USA): GAPDH (#D16H11), MDA5 (#D74E4), RIG-I (#D14G6) and TLR3 (#D10F10), all at 1:1000 dilution in TBST + 5% BSA (20 mM TRIS-base, 137 mM NaCl, 0.1% Tween 20). Membranes were washed 3 × 15 min in TBST and incubated 1 h at room temperature with an HRP-conjugated secondary anti-rabbit goat antibody (1:5000 in TBST + 5% BSA)(#09/2029)(Cell Signaling), followed by a second wash of 3 × 15 min in TBST and 5 min incubation with Clarity Max Western ECL Substrate (Bio-Rad). Optical density was quantified by a LI-COR odyssey Fc imager (LI-COR, Lincoln, USA) and the Image Studio Lite software (LI-COR), and optical ratios of target protein and GAPDH were calculated for each sample. For each membrane, TLR3 was first detected. Membranes were then stripped with Restore PLUS western blot stripping buffer (Thermo Scientific), blocked with EveryBlot blocking buffer and incubated with primary antibody against MDA5. This procedure was repeated for RIG-I. All samples were then normalized to the mean target/GAPDH ratio for each saline group.

### Statistical analysis

All statistical analysis was performed using GraphPad Prism version 9 (GraphPad software, San Diego, USA). Data are presented as mean ± standard deviation (SD). Multiple comparisons of all groups (strain and treatment) were made using a 2way ANOVA with Tukey’s multiple comparisons test. *P*-values of < 0.05 were considered statistically significant.

## Results

### Intranasal administration of dsRNA increases lung inflammation in BALB/c and C57Bl/6J, but not C57Bl/6N mice

**Fig. 2:**
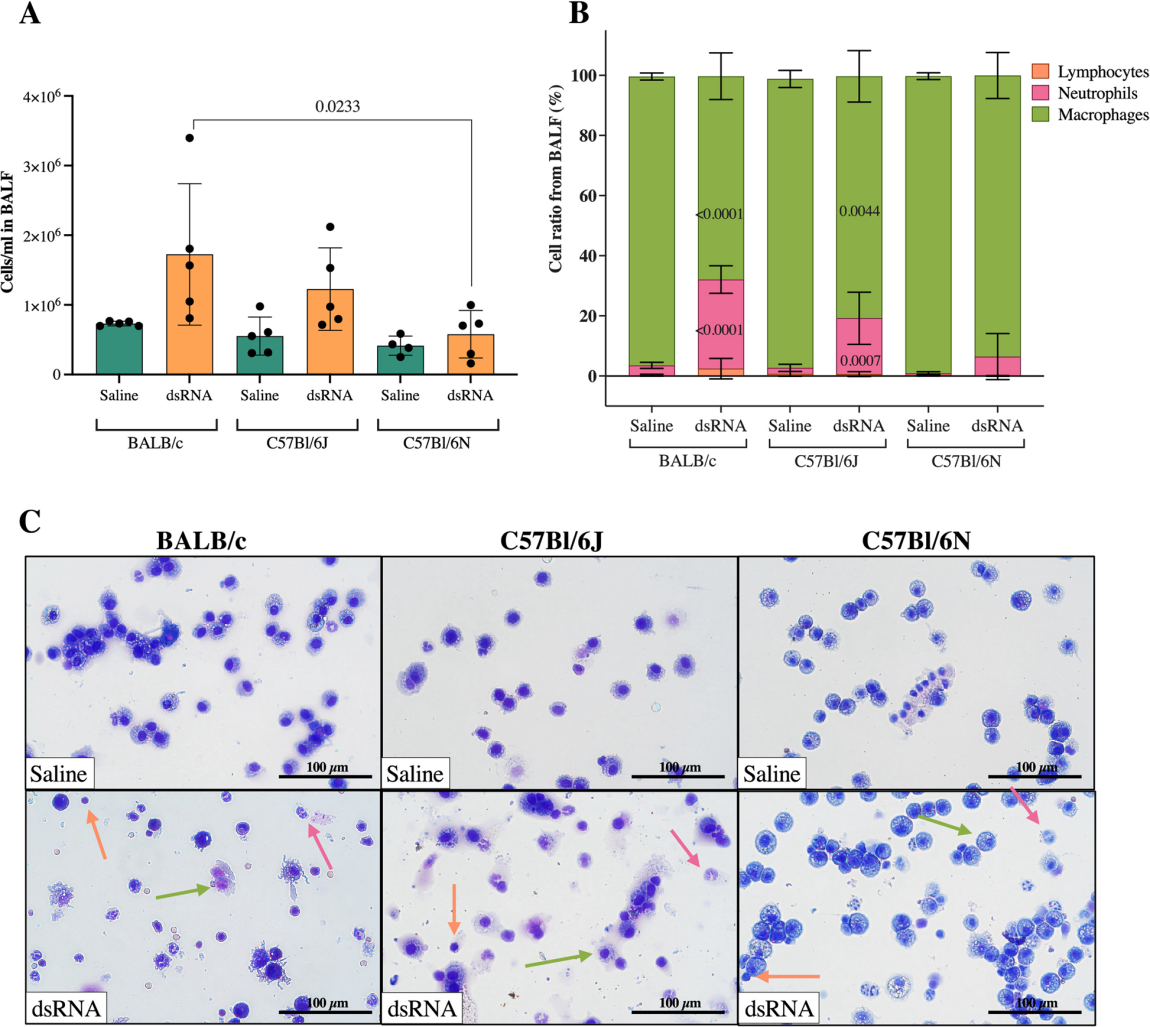
Effect of i.n. dsRNA administration on BALF cellular infiltration in mice. dsRNA (100 µg) or saline were i.n. administered to C57Bl/6J, C57Bl/6N or BALB/c mice once per day for three consecutive days, and cell concentration (cells/ml) (**A**) and cellular composition (%) (**B**) were determined in BALF. Representative pictures of BALF composition are shown in (**C**) (100X magnification). Orange arrows depict lymphocytes, purple arrows depict neutrophils and green arrows depict macrophages. Scale bars indicate 100 µm. Data are presented as mean ± SD. Differences between groups were tested using a 2way ANOVA with Tukey’s multiple comparisons test. *P*-values of < 0.05 are indicted in the graphs, each data point represents one mouse from one experiment (*n* = 4–5 per group)

To investigate the inflammatory response to dsRNA of BALB/c, C57Bl/6J and C57Bl/6N mice, 100 µg dsRNA (poly(I:C)) or saline were administered i.n. once per day for three consecutive days (Fig. 1). To assess the degree of inflammation in the lung, cell concentration and composition was determined by an automated cell counter and subsequential differential cell count of BALF cells, respectively. There was a trend towards an increase in the concentration of cells in response to dsRNA in BALB/c (*p* = 0.0632), but not in C57Bl/6J or C57Bl/6N mice (Fig. 2a). A key feature of the lung inflammatory response to viral infections is neutrophil infiltration [24]. Here, differential cell count revealed a significant increase in the percentage of neutrophils in response to dsRNA in BALB/c and C57Bl/6J mice, while only a modest (non-significant) increase in neutrophil percentage was seen in C57Bl/6N (Fig. 2b-c), suggesting a reduced capacity of C57Bl/6N to respond to viral stimuli. The same pattern was found when determining the total number of neutrophils in the BALF from each strain (Additional file 5b). No changes in lymphocyte percentages or total numbers in any strain (Fig. 2b-c, Additional file 5a) were found.

### Total protein concentration and LDH activity in BALF are increased in response to dsRNA in BALB/c and C57Bl/6J mice, but not C57Bl/6N mice

**Fig. 3:**
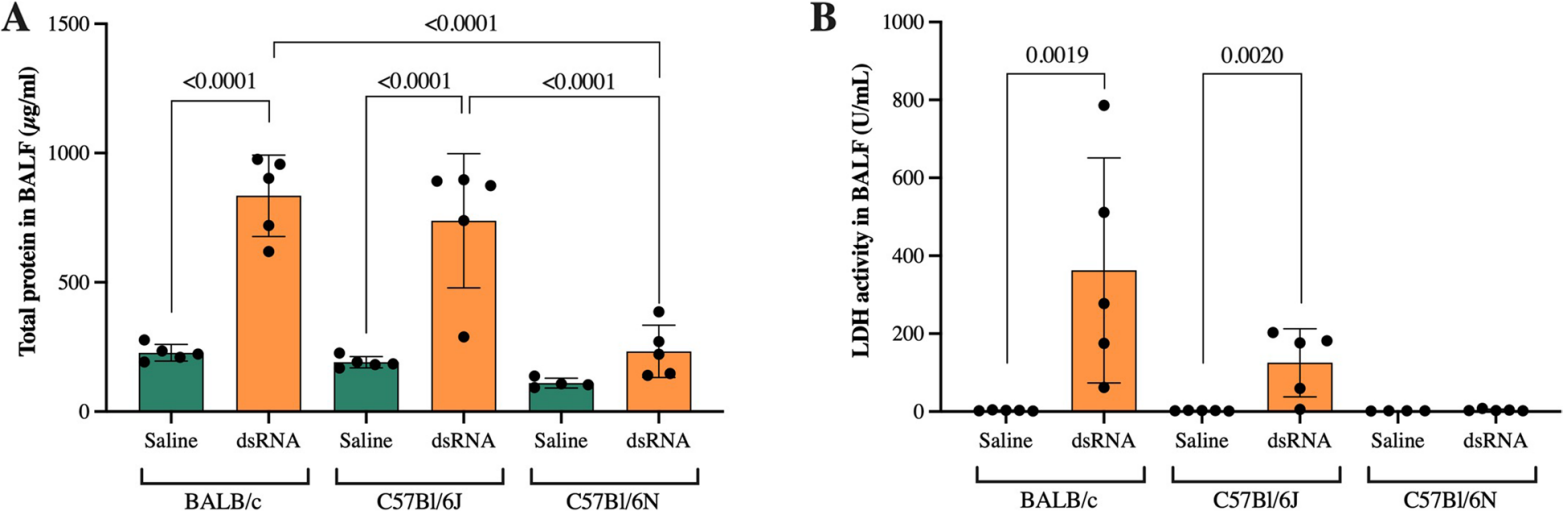
Total protein concentration and LDH activity in BALF after i.n. dsRNA administration in mice. Total protein concentration (**A**) and LDH activity (**B**) in BALF were measured after 3 days of i.n. administration of 100 µg dsRNA or saline to BALB/c, C57Bl/6J and C57Bl/6N mice. Data are presented as mean ± SD. Differences between groups were tested using a 2way ANOVA with Tukey’s multiple comparisons test. *P*-values of < 0.05 are indicted in the graphs, each data point represents one mouse from one experiment (*n* = 4–5 per group)

Increases in total protein concentration is a feature of lung inflammation in mice [23]. dsRNA administration markedly increased total protein concentration about fourfold in both BALB/c and C57Bl/6J mice (Fig. 3a). However, only a modest increase (~ twofold) was observed for C57Bl/6N mice, that did not reach statistical significance (Fig. 3a). Indeed, the increases in total protein concentration in response to dsRNA in BALB/c and C57Bl/6J mice were both significantly larger when compared to the dsRNA-induced increase in C57Bl/6N mice (Fig. 3a).

The activity of lactate dehydrogenase (LDH) in BALF is a marker of tissue damage which can be seen in inflammation, and is known to increase in viral-induced asthma exacerbations [25]. Here, dsRNA administration evoked increased LDH activity in BALB/c and C57Bl/6J, but not C57Bl/6N mice (Fig. 3b).

### PRR expression is induced in response to dsRNA administration in BALB/c and C57Bl/6J mice, but not C57Bl/6N mice

**Fig. 4:**
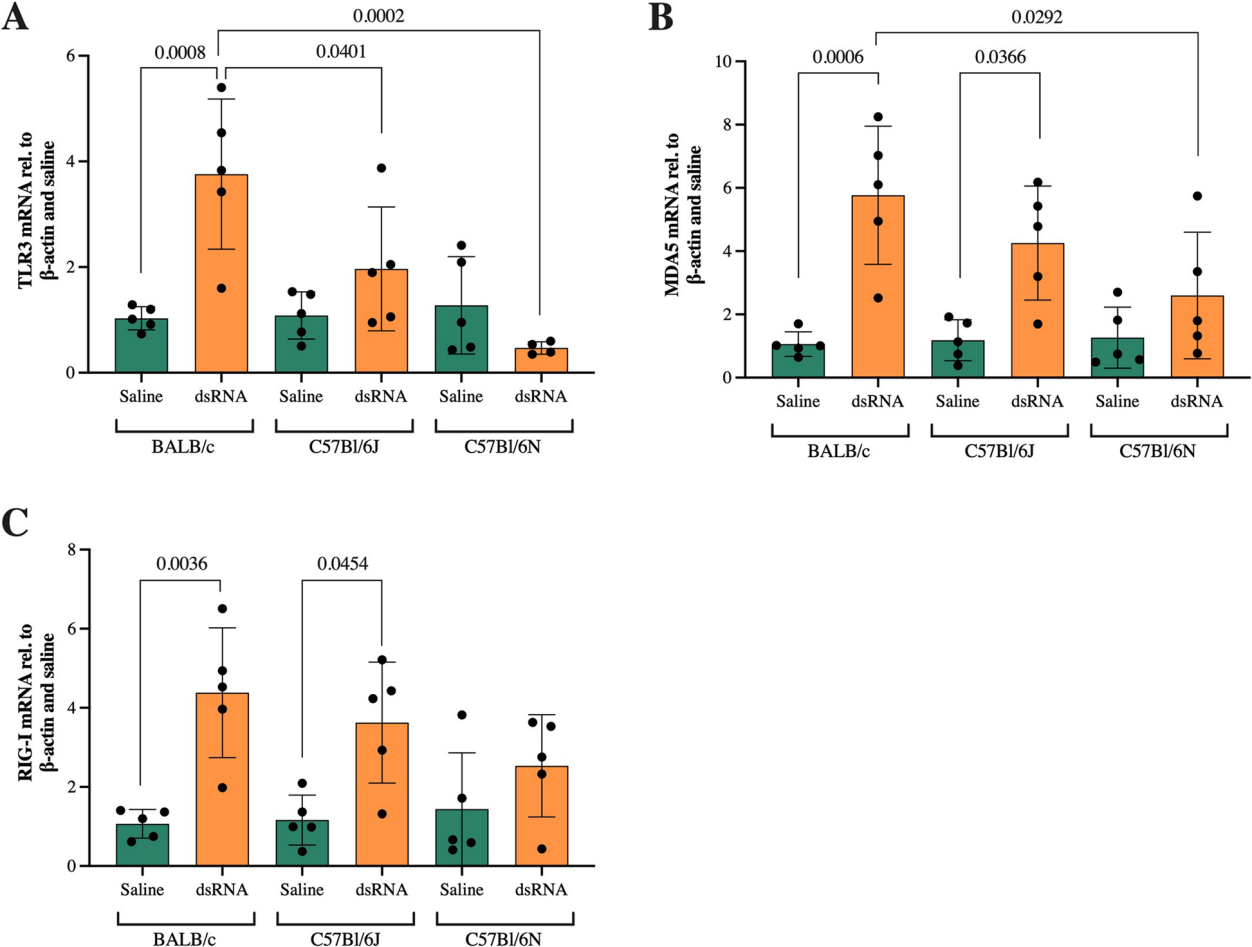
The effect of i.n. dsRNA administration on gene expression of TLR3, MDA5 and RIG-I. dsRNA (100 µg) or saline were i.n. administered to BALB/c, C57Bl/6J or C57Bl/6N mice once per day for three consecutive days, and gene expression of TLR3 (**A**), MDA5 (**B**), and RIG-I (**C**) was determined by RT-qPCR in lung homogenates. Data are presented as mean ± SD, as relative to reference gene β-actin and fold change compared to the mean of saline within each strain. Differences between groups were tested using a 2way ANOVA with Tukey’s multiple comparisons test. *P*-values of < 0.05 are indicted in the graphs, each data point represents one mouse from one experiment (*n* = 5 per group)

As part of the innate immune system, PRRs in the lung sense virus components to orchestrate anti-viral and inflammatory responses [6]. Endosomal TLR3 and cytosolic receptors MDA5 and RIG-I sense viral dsRNA, the replication intermediate of ssRNA viruses [5]. Thus, we compared the induction of PRR expression in response to dsRNA in the three mouse strains. TLR3 mRNA was significantly upregulated in response to dsRNA in BALB/c mice (fourfold compared to saline control), but not in the other two strains (Fig. 4a). Furthermore, the induction of TLR3 gene expression in dsRNA-challenged mice was significantly greater in BALB/c compared to C57Bl/6J and C57Bl/6N (Fig. 4a). Similarly, MDA5 and RIG-I mRNA were significantly upregulated (6 and fourfold compared to saline control) in BALB/c mice in response to dsRNA (Fig. 4b-c). Interestingly, dsRNA administration evoked similar MDA5 and RIG-I responses also in C57Bl/6J (Fig. 4b-c). No significant upregulations in response to dsRNA were observed in C57Bl/6N mice (Fig. 4b-c). Moreover, the dsRNA-induced increase of MDA5 gene expression in BALB/c mice was greater compared to that of C57Bl/6N (Fig. 4b).

**Fig. 5:**
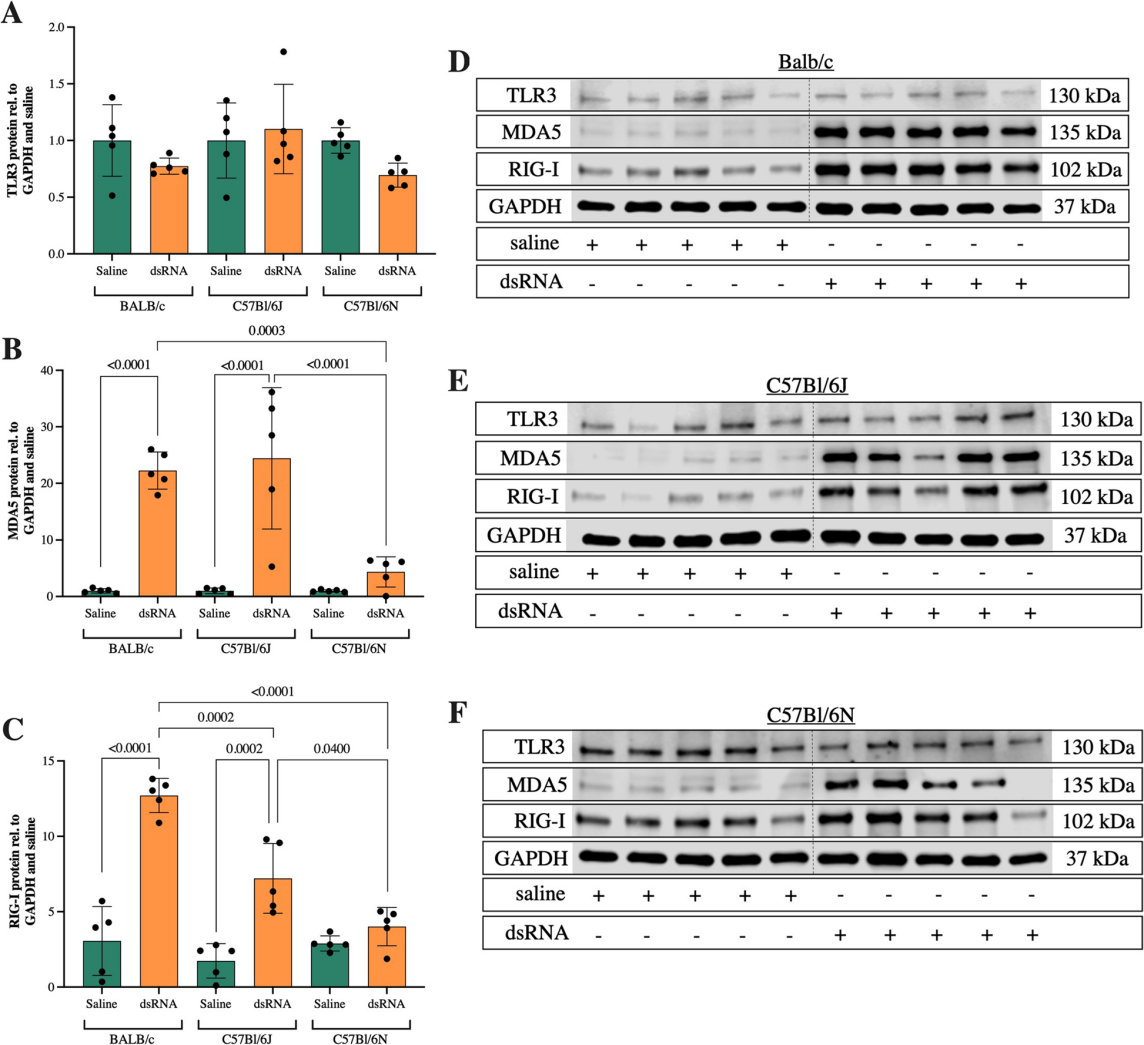
The effect of i.n. dsRNA administration on protein expression of TLR3, MDA5 and RIG-I. dsRNA (100 µg) or saline were i.n. administered to BALB/c, C57Bl/6J or C57Bl/6N mice once per day for three consecutive days and protein expression of PRRs in lung homogenates was analyzed by western blot. Quantification of immunoblots was performed for TLR3 (**A**), MDA5 (**B**) and RIG-I (**C**). Cropped immunoblots are presented for each of the strains; BALB/c (**D**), C57Bl/6J (**E**) and C57Bl/6N (**F**). Data are presented as mean ± SD, as relative to GAPDH and fold change compared to the mean of saline within each strain. Differences between groups were tested using a 2way ANOVA with Tukey’s multiple comparisons test. *P*-values of < 0.05 are indicted in the graphs, each data point represents one mouse from one experiment (*n* = 5 per group)

On protein level, the TLR3 expression was surprisingly unaffected by dsRNA administration in all strains (Fig. 5a, d-f, Additional files 1 and 2). However, comparable to its mRNA levels, protein expression of MDA5 was upregulated in response to dsRNA in BALB/c and C57Bl/6J mice (20-fold compared to saline control) (Fig. 5b, d-e, Additional files 1 and 3). C57Bl/6N mice showed a small (fourfold compared to saline control), non-significant increase of MDA5 protein in response to dsRNA, and this increase was significantly lower compared to that of BALB/c and C57Bl/6J mice (Fig. 5b, f).

The RIG-I protein expression significantly increased in BALB/c (13-fold compared to saline control) and C57Bl/6J (sevenfold compared to saline control) but not in C57Bl/6N mice after dsRNA administration (Fig. 5c, d-f, Additional file 1 and 4). Here, the dsRNA response in BALB/c was significantly higher compared to both C57Bl/6J and C57Bl/6N mice, and the C57Bl/6J response was in turn significantly larger than the C57Bl/6N (Fig. 5c).

### BALB/c and C57Bl/6J mice, but not C57Bl/6N, respond with pro-inflammatory cytokine expression after dsRNA administration

**Fig. 6:**
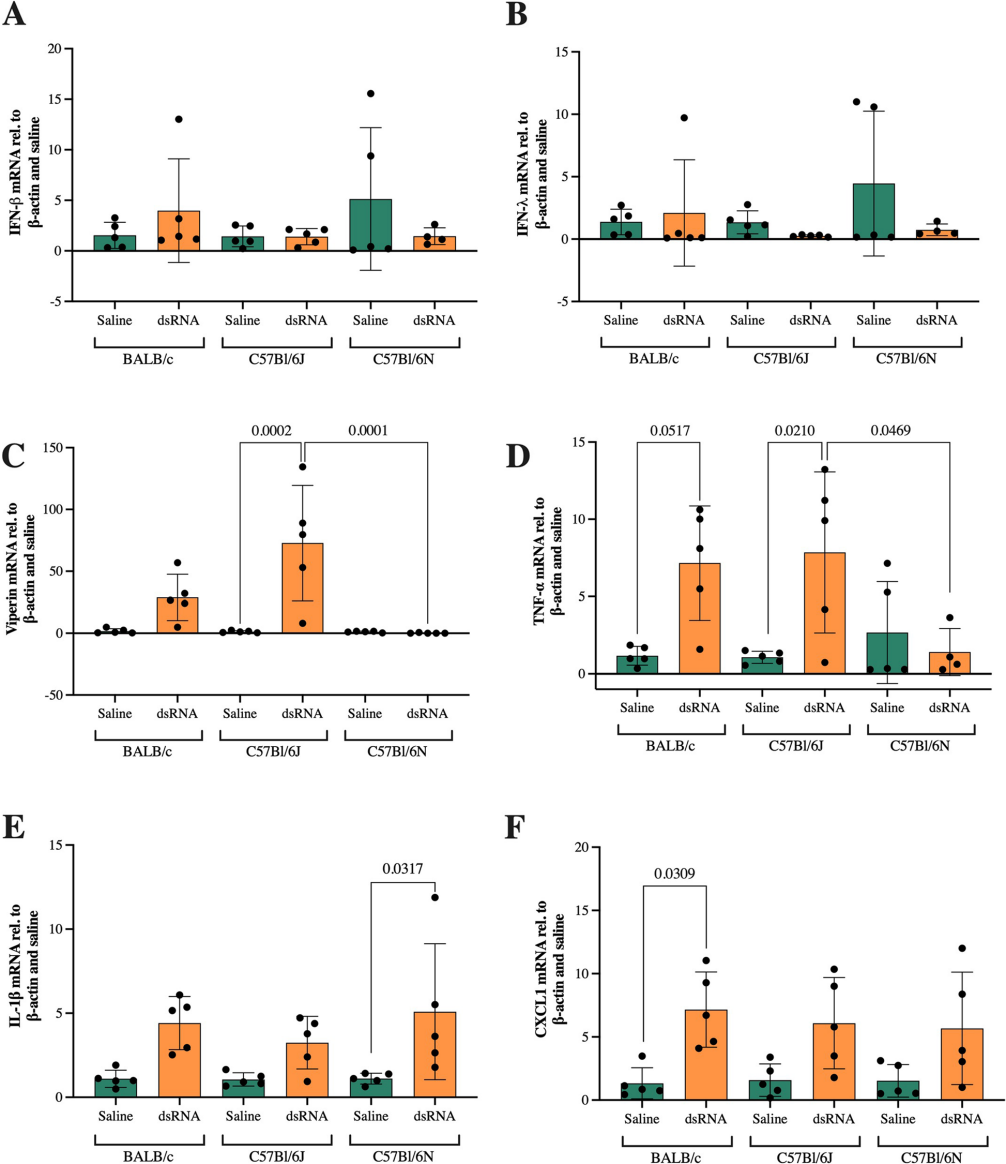
Gene expression of IFN-β, IFN-λ, viperin, TNF-α, IL-1β and CXCL1 after i.n. dsRNA administration. dsRNA (100 µg) or saline were i.n. administered to BALB/c, C57Bl/6J or C57Bl/6N mice once per day for three consecutive days and gene expression of IFN-β (**A**), IFN-λ (**B**), viperin (**C**), TNF-α (**D**), IL-1β (**E**) and CXCL1 (**F**) was determined by RT-qPCR in lung homogenates. Data are presented as mean ± SD, as relative to the reference gene β-actin and fold change compared to the mean of saline controls within each strain. Differences between groups were tested using a 2way ANOVA with Tukey’s multiple comparisons test. *P*-values of < 0.05 are indicted in the graphs, each data point represents one mouse from one experiment (*n *= 5 per group)

As a result of PRR sensing of dsRNA, production of anti-viral interferons and pro-inflammatory cytokines is initiated in infected cells [6]. Hence, as a measure of the immunological response to dsRNA, we analyzed the gene expression of IFN-β, IFN-λ, the IFN-stimulated gene viperin and pro-inflammatory TNF-α, IL-1β and CXCL1. Compared to saline controls, dsRNA administration did not evoke any IFN-β or IFN-λ responses, as evidenced by unchanged mRNA expression in all strains (Fig. 6a-b). In contrast, C57Bl/6J mice showed a significant induction of the IFN-stimulated gene viperin in response to dsRNA administration (73-fold compared to saline controls) (Fig. 6c). Furthermore, BALB/c mice responded with a mean 29-fold induction of viperin to dsRNA, however, this increase was not statistically significant (*p* = 0.3241). BALB/c and C57Bl/6J mice responded with increased mRNA levels of TNF-α to dsRNA administration (7 and eightfold compared to saline controls, respectively), where the C57Bl/6J response was also significantly larger compared to the C57Bl/6N response (Fig. 6d). Surprisingly, C57Bl/6N was the only strain that significantly increased gene expression of IL-1β in response to dsRNA (fivefold compared to saline control) (Fig. 6e). BALB/c mice also responded with an increased IL-1β expression (fourfold compared to saline control), but this failed to reach significance (*p *= 0.1017) (Fig. 6e). Moreover, only BALB/c mice responded with significantly elevated levels of CXCL1 mRNA in response to dsRNA (sevenfold compared to saline controls) (Fig. 6f).

**Fig. 7:**
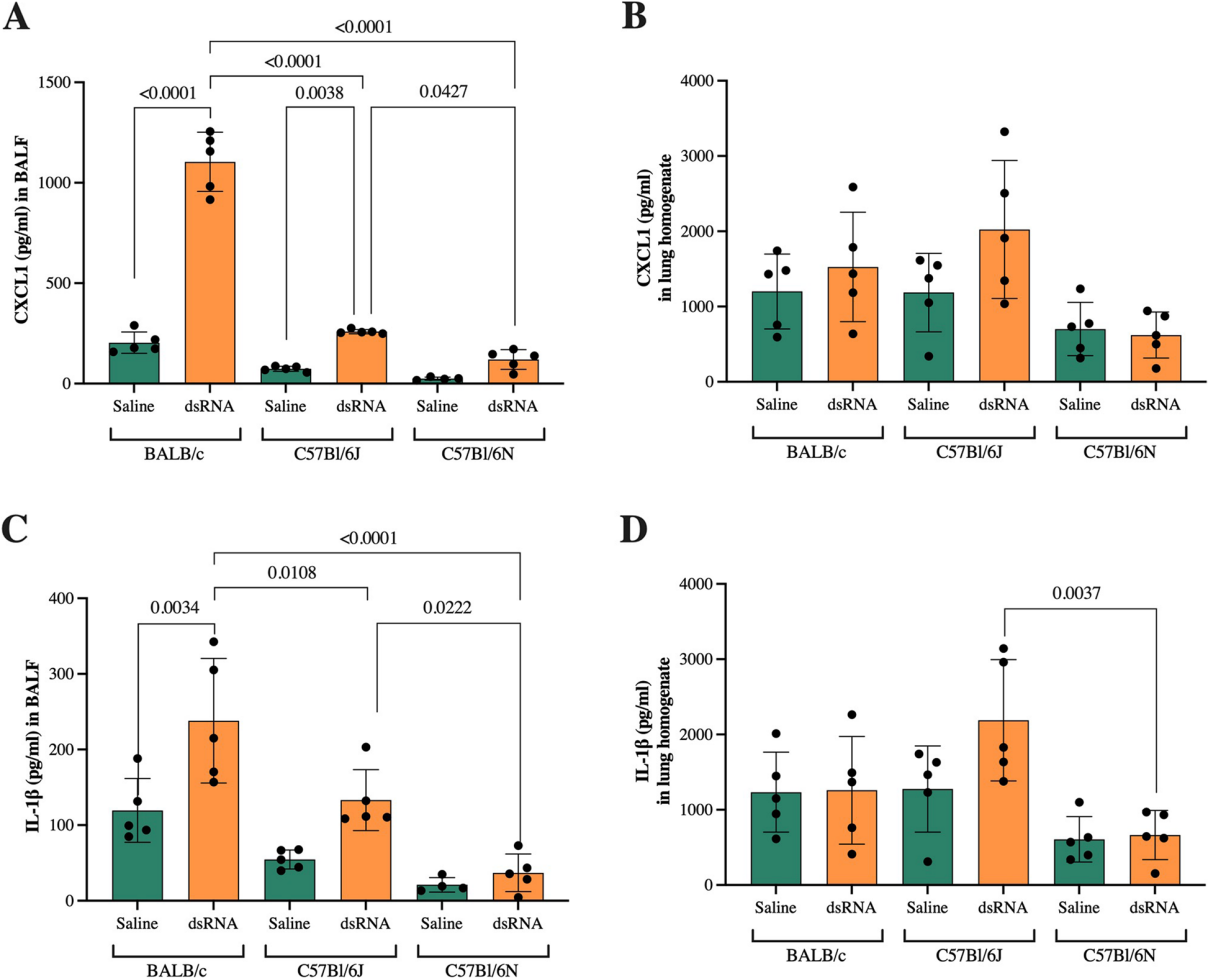
Protein expression of IL-1β and CXCL1 after i.n. dsRNA administration. dsRNA (100µg) or saline were i.n. administered to BALB/c, C57Bl/6J or C57Bl/6N mice once per day for three consecutive days, and protein expression of CXCL1 (**A**) and IL-1β (**C**) in BALF and CXCL1 (**B**) and IL-1β (**D**) in lung homogenates was determined by ELISA. Data are presented as pg/ml and mean ± SD. Differences between groups were tested using a 2way ANOVA with Tukey’s multiple comparisons test. *P*-values of < 0.05 are indicted in the graphs, each data point represents one mouse from one experiment (*n* = 5 per group)

To confirm gene expression results, we analyzed the protein expression of CXCL1 and IL-1β in both BALF and lung homogenates. In BALF, CXCL1 protein expression was significantly increased in response to dsRNA in BALB/c and C57Bl/6J strains (5 and threefold compared to saline control, respectively), with a significantly greater response in BALB/c mice compared to both C57Bl/6J and C57Bl/6N (Fig. 7a). In addition, the protein levels of CXCL1 after dsRNA administration were significantly higher in C57Bl/6J compared to C57Bl/6N mice (Fig. 7a). In lung homogenates, no significant differences in response to dsRNA administration were detected (Fig. 7b).

IL-1β expression in BALF was significantly increased after dsRNA administration in BALB/c (twofold compared to saline control), and increased (*p* = 0.0864) in C57Bl/6J mice (twofold compared to saline control) (Fig. 7c). Contrary to its gene expression, dsRNA administration did not increase protein expression of IL-1β in C57Bl/6N mice (Fig. 7c). The dsRNA response of BALB/c was significantly greater compared to both C57Bl/6J and C57Bl/6N, and the C57Bl/6J response was greater than the C57Bl/6N response (Fig. 7c). No significant changes were found in lung homogenates within strains, but the dsRNA response in C57Bl/6J was significantly larger compared to the C57Bl/6N response (Fig. 7d).

## Discussion

In this study, we compared the respiratory innate immune response to i.n. administration of dsRNA of BALB/c, C57Bl/6J and C57Bl/6N mice. We demonstrate that both BALB/c and C57Bl/6J mice display distinct upregulation of inflammatory markers in response to dsRNA, while the inflammatory response of C56Bl/6N mice is attenuated. In general, comparing the dsRNA response between strains, the BALB/c strain shows the most prominent inflammatory response. These data reveal distinct strain differences of the pulmonary immune response to a viral mimic and underscore the importance of strain selection when designing mouse studies of respiratory viral infections.

Immunological differences between C57Bl/6 and BALB/c mice have been previously reported. In a study by Watkiss et al., the immune responses toward the ssRNA pneumonia virus of mice (PVM) in BALB/c and C57Bl/6 (substrain not specified) mice were compared [15]. Watkiss et al. show that the BALB/c strain responds with higher levels of CXCL8/CXCL1 compared to the C57Bl/6 strain. In addition, they conclude that the immune response of BALB/c mice is both earlier and stronger compared to C57Bl/6 [15]. Our study, showing a larger immune response of BALB/c mice after 3 days of dsRNA compared to both C57Bl/6N and C57/Bl/6 J, supports these observations. Furthermore, in a study by Yasui et al., the immune response to SARS-CoV-2 infection was compared between BALB/c and C57Bl/6J mice. In analyzes of lung homogenates 3 days post infection, BALB/c mice had higher protein levels of IP-10, IL-1β, TNF-α, M-CSF, IL-4, IL-12, IL-15 and IL-17 [14]. In contrast, in none of our lung homogenate analyzes (protein or mRNA), we were able to detect any differences in cytokine expression between the BALB/c and C57Bl/6J strains after dsRNA administration. The most marked differences we found were in BALF, where BALB/c had a higher concentration of IL-1β and CXCL1 after dsRNA administration compared to C57Bl/6J. Nonetheless, our overall conclusion agrees with Yasui et al.: BALB/c mice exhibit a more pronounced inflammation compared to C57Bl/6J mice in response to viral stimuli. Finally, C57Bl/6 mice have been shown to have a much weaker immune reactivity of the lung towards allergens and development of asthma-like features compared to BALB/c mice [26]. This, together with a stronger response to viral stimulation, the main trigger of asthma exacerbations, makes BALB/c mice a suitable candidate for developing asthma exacerbation models.

C57Bl/6 mice are widely used in the field of molecular biology and biomedicine due to the extensive availability of commercial genetically modified mice, their easy breeding, robustness and availability of congenic strains. Despite several studies investigating the differences of viral-induced immune responses in BALB/c and C57Bl/6 mice, to our knowledge, this is the first study to compare the dsRNA-induced pulmonary immune response of two substrains of C57Bl/6: the C57Bl/6N and the C57Bl/6J. Our results show significant differences of general inflammation markers in response to dsRNA: the J-strain exhibits higher concentration of total protein, CXCL1 and IL-1β protein levels in BALF, as well as larger increase in MDA5 and RIG-I protein expression and TNF-α gene expression in lung homogenates compared to the N-strain. A study by Warden et al. describes the inflammatory response of the brain of C57Bl/6J and C57Bl/6N mice at 3 and 24 h post intraperitoneal injection of dsRNA. Similar to our results, at 24 h, the response of C57Bl/6J was more pronounced than that of C57Bl/6N. However, at 3 h post dsRNA administration, inflammatory gene expression was induced to a similar extent in both strains. Thus, they conclude that the innate immune response of C57Bl/6N mice is shorter compared to C57Bl/6J [20]. Therefore, the discrepancies observed in our study could potentially be explained by the kinetics of the inflammatory response, and should be addressed in further studies.

In genetic analyses of the C57Bl/6J and N- mouse strains, several SNPs and structural variants have been identified [16, 27]. Indeed, Bezdek et al. have reported differences in the development of Aldara-induced psoriasiform dermatitis (AIPD) between C57Bl/6J and C57Bl/6N. Aldara is a TLR7 agonist, applied to the skin to induce skin inflammation. In agreement with our findings, Bezdek et al. show that the C57Bl/6J strain developed more severe skin inflammation than C57Bl/6N [28]. Of particular interest for these and our observations is an in-frame five-exon deletion of the nicotinamide nucleotide transhydrogenase (nnt) gene in C57Bl/6J mice, leading to a loss of the nnt protein [29]. Nnt over expression in murine macrophages suppresses reactive oxygen species (ROS) and cytokine release. In addition, transgenic C57Bl/6J mice expressing nnt produce less IL-1β, IL-6 and CXCL1 and are more vulnerable to *S. pneumoniae* pulmonary infection compared to WT C57Bl/6J mice [17]. To our knowledge, there has been no investigations on the relationship between nnt and PRRs such as TLR7, TLR3, MDA5 and RIG-I, but in the light of our results, this is something that would be interesting to investigate in further studies.

PRRs are sensors of viral components, and orchestrate immune responses. In regards of dsRNA, TLR3 is responsible for the initial recognition and signaling, while cytosolic MDA5 and RIG-I are important for later responses. In a study employing RV infection of human bronchial epithelial cells, TLR3 was constitutively expressed while MDA5 and RIG-I were induced later during infection in response to TLR3 and IFN signaling [30]. In accordance with these observations, although TLR3 mRNA was significantly induced in BALB/c mice, we found no induction of TLR3 protein expression upon dsRNA administration in any of the investigated mouse strains. In contrast, MDA5 and RIG-I mRNA and protein levels were upregulated in response to dsRNA in BALB/c and C57Bl/6J mice. In terms of protein analysis, BALB/c and C57Bl/6J had a significantly larger increase of MDA5 and RIG-I in response to dsRNA compared to the C57Bl/6N strain. Thus, our findings suggest that TLR3 downstream signaling might be restricted in C57Bl/6N mice. Further studies are warranted to identify the underlying cause and the role of TLR3 in the attenuated response of C57Bl/6N mice.

We failed to detect any significant induction of IFN-β and IFN-λ by dsRNA in any strain. However, the IFN-stimulated gene viperin was significantly induced by dsRNA administration in C57Bl/6J mice. This could be explained by the rapid nature of the IFN response. In fact, in the study by Warden et al., the dsRNA-induced IFN-β expression in the brain was highest at 3 h post dsRNA challenge in C57Bl/6J mice [20]. Thus, the increase in the IFN-stimulated genes viperin, but also MDA5 and RIG-I, are probably secondary effects of IFN expression at earlier time-points.

Both a strength and limitation of the present study is the use of dsRNA as a viral mimic. The strength lies in the reproducibility and uniformity of dsRNA use, as the “pathogen burden” can be better controlled compared to real viral infection. This eliminates confounding factors of viral susceptibility and variation in pathogen load when merely analyzing the immune response of mice. The limitation covers the fact that different viruses evoke different immune responses. For instance, infection of human bronchial epithelial cells with HRV-C has been shown to induce a lower cytokine release of CCL5, MCP-1 and IL-8 compared to infection with RSV [31]. Hence, dsRNA is a suitable viral mimic for some, but perhaps not all ssRNA viruses. As a consequence, it is of importance to be aware of this limitation when selecting mouse strains for future research.

## Conclusions

Our study highlights remarkable discrepancies in the pulmonary innate immune responses towards dsRNA of BALB/c, C57Bl/6J and C57Bl/6N mice. Altogether, our results indicate that C57Bl/6N have an attenuated inflammatory response to dsRNA and that BALB/c present the strongest pro-inflammatory response to dsRNA. C57Bl/6J mice present similar features as BALB/c mice, although slightly less pronounced. Especially for C57Bl6/N and C57Bl/6J, one must be aware of the genetic background when comparing studies, as false results might be a consequence of different genetic backgrounds. Similarly, results might be hard to validate if different substrains are used. Overall, we suggest that the present results contribute to important progress in research on respiratory viral infections.

## Supplementary Information

**Additional file 1: Additional figure 1.** Uncropped immunoblots of GAPDH blots. Chemiluminescence channel of C57Bl/6J blot (A), BALB/c blot (C) and C57Bl/6N blot (E). Chemiluminescence and 700 nm channel merge of C57Bl/6J blot (B), BALB/c blot (D) and C57Bl/6N (F), to visualize ladder. Ladder size is indicated.

**Additional file 2: Additional figure 2.** Uncropped immunoblots of TLR3 blots. Chemiluminescence channel of C57Bl/6J blot (A), BALB/c blot (C) and C57Bl/6N blot (E). Chemiluminescence and 700 nm channel merge of C57Bl/6J blot (B), BALB/c blot (D) and C57Bl/6N (F), to visualize ladder. Ladder size is indicated.

**Additional file 3:** **Additional figure 3.** Uncropped immunoblots of MDA5 blots. Chemiluminescence channel of C57Bl/6J blot (A), BALB/c blot (C) and C57Bl/6N blot (E). Chemiluminescence and 700 nm channel merge of C57Bl/6J blot (B), BALB/c blot (D) and C57Bl/6N (F), to visualize ladder. Ladder size is indicated.

**Additional file 4:** **Additional figure 4.** Uncropped immunoblots of RIG-I blots. Chemiluminescence channel of C57Bl/6J blot (A), BALB/c blot (C) and C57Bl/6N blot (E). Chemiluminescence and 700 nm channel merge of C57Bl/6J blot (B), BALB/c blot (D) and C57Bl/6N (F), to visualize ladder. Ladder size is indicated.

**Additional file 5: Additional figure 5.** Effect of i.n. dsRNA administration on BALF cellular infiltration in mice. dsRNA (100 µg) or saline were i.n. administered to C57Bl/6J, C57Bl/6N or BALB/c mice once per day for three consecutive days, and the total number of lymphocytes (A), neutrophils (B) and macrophages (C) were determined in BALF using cytospin, differential cell counting and cell concentration measurements. Data are presented as mean ± SD. Differences between groups were tested using a 2way ANOVA with Tukey’s multiple comparisons test. *P*-values of <0.05 are indicted in the graphs, each data point represents one mouse from one experiment (*n *= 4-5).

## Data Availability

Data sharing is not applicable to this article as no datasets were generated or analyzed during the current study.

## Abbreviations

AIPD: Aldara-induced psoriasiform dermatitis
BALF: Bronchoalveolar lavage fluid
CXCL: C-X-C motif chemokine ligand
dsRNA: Double stranded RNA
IL: Interleukin
IFN: Interferon
LDH: Lactate dehydrogenase
LRTI: Lower respiratory tract infections
MDA5: Melanoma differentiation-associated protein 5
NNT: Nicotinamide nucleotide transhydrogenase
PRR: Pattern recognition receptor
RIG-I: Retinoic acid-inducible gene I
ROS: Reactive oxygen species
RSV: Respiratory syncytial virus
RV: Rhinovirus
ssRNA: Single stranded RNA
TLR: Toll-like receptor
TNF: Tumor necrosis factor
