# Supplementary material for: C57Bl/6N mice have an attenuated lung inflammatory response to dsRNA compared to C57Bl/6J and BALB/c mice
Source: J Inflamm (Lond). 2023 Feb 21;20:6. doi: 10.1186/s12950-023-00331-4 (PMC9942641; doi:10.1186/s12950-023-00331-4)
Supplement: Supplementary file 1 — Additional file 1: Additional figure 1. Uncropped immunoblots of GAPDH blots. Chemiluminescence channel of C57Bl/6J blot (A), BALB/c blot (C) and C57Bl/6N blot (E). Chemiluminescence and 700 nm channel merge of C57Bl/6J blot (B), BALB/c blot (D) and C57Bl/6N (F), to visualize ladder. Ladder size is indicated. [file 12950_2023_331_MOESM1_ESM.docx]

**Additional file 1**


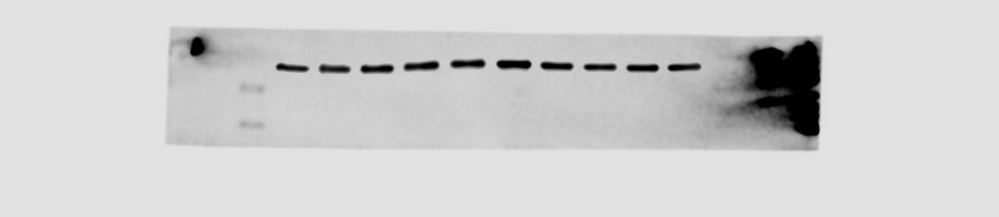

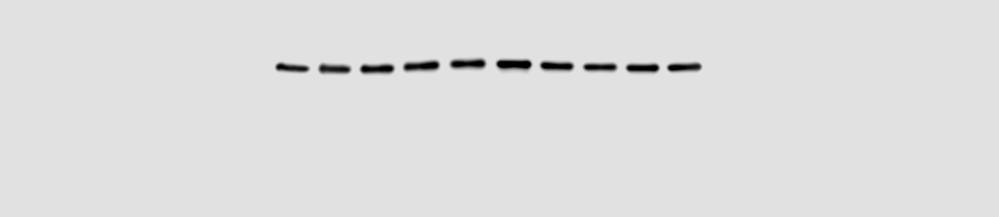

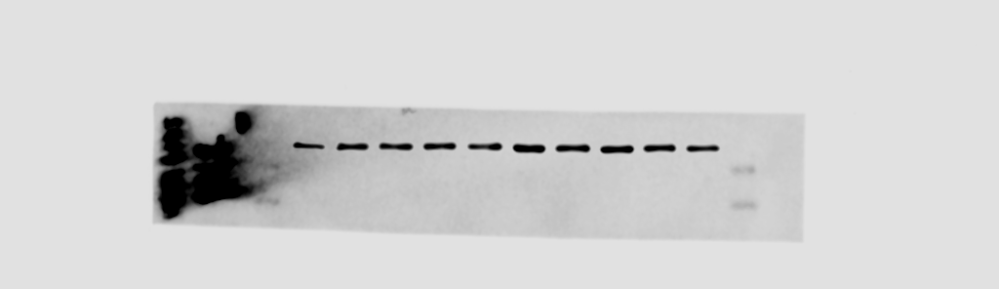

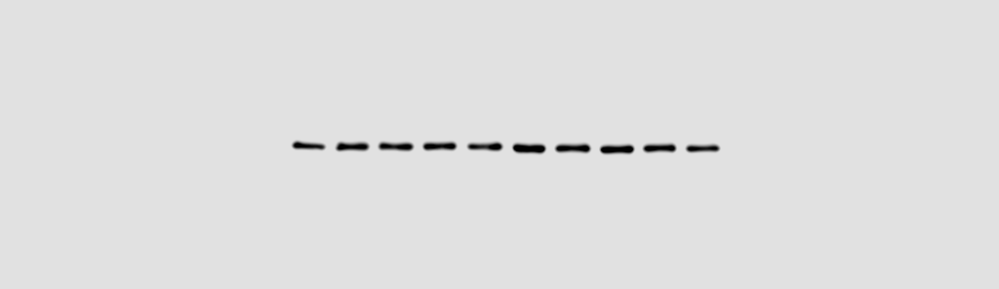

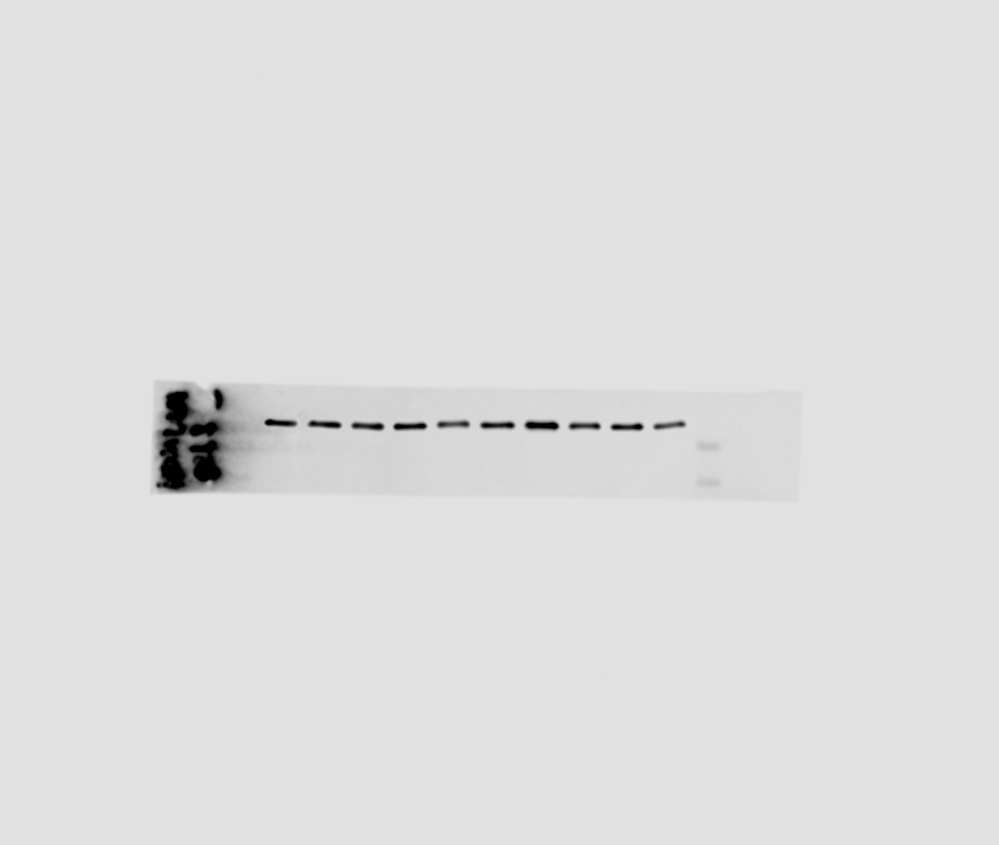

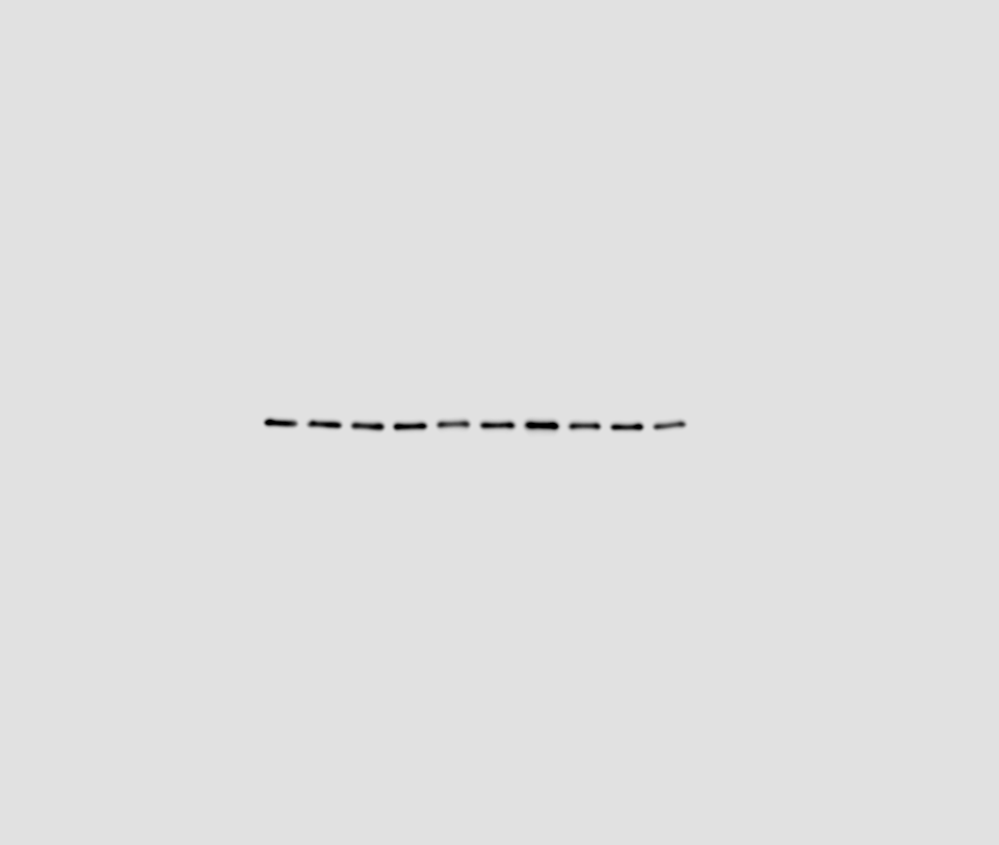


A

B

C

D

E

F

**Additional figure 1.** **Uncropped immunoblots of GAPDH blots.** Chemiluminescence channel of C57Bl/6J blot (A), BALB/c blot (C) and C57Bl/6N blot (E). Chemiluminescence and 700 nm channel merge of C57Bl/6J blot (B), BALB/c blot (D) and C57Bl/6N (F), to visualize ladder. Ladder size is indicated.

30 kDa

25 kDa

30 kDa

25 kDa

30 kDa

25 kDa
