# Supplementary material for: C57Bl/6N mice have an attenuated lung inflammatory response to dsRNA compared to C57Bl/6J and BALB/c mice
Source: J Inflamm (Lond). 2023 Feb 21;20:6. doi: 10.1186/s12950-023-00331-4 (PMC9942641; doi:10.1186/s12950-023-00331-4)
Supplement: Supplementary file 4 — Additional file 4: Additional figure 4. Uncropped immunoblots of RIG-I blots. Chemiluminescence channel of C57Bl/6J blot (A), BALB/c blot (C) and C57Bl/6N blot (E). Chemiluminescence and 700 nm channel merge of C57Bl/6J blot (B), BALB/c blot (D) and C57Bl/6N (F), to visualize ladder. Ladder size is indicated. [file 12950_2023_331_MOESM4_ESM.docx]

**Additional file 4**


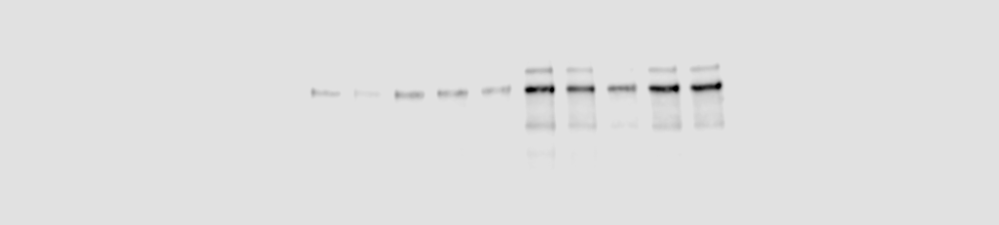

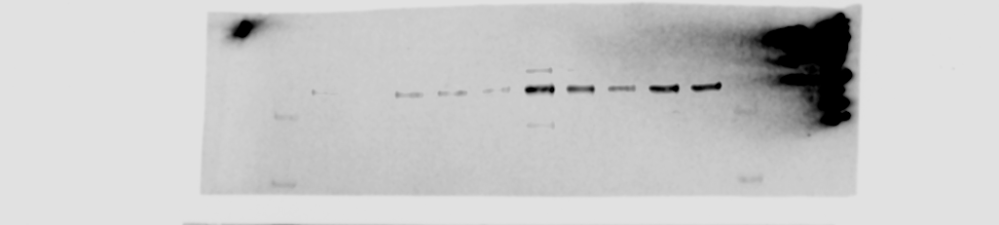

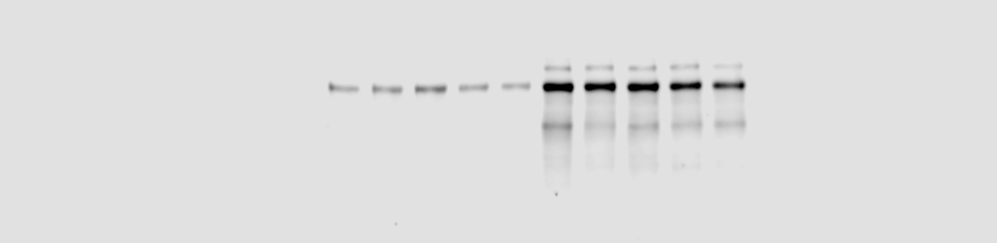

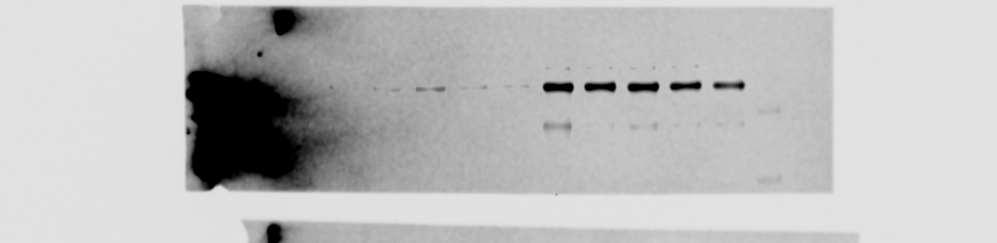

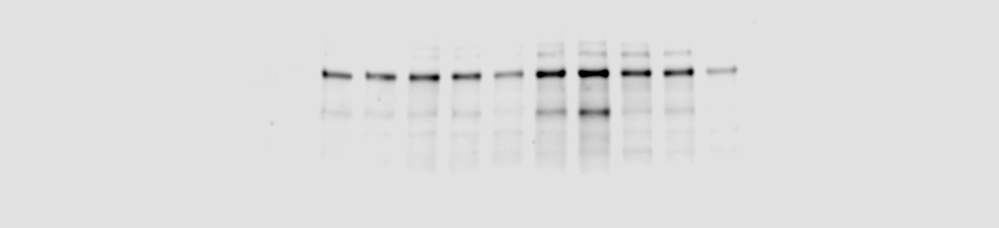

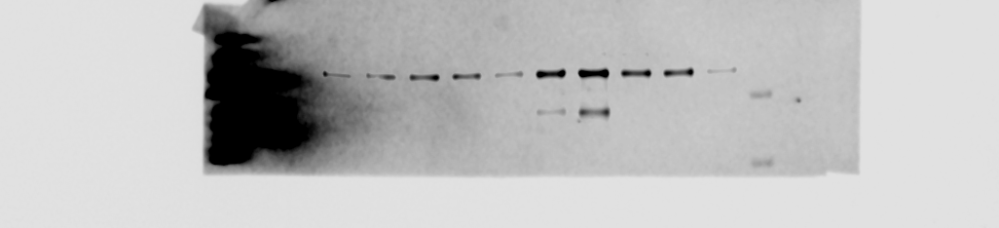


F

A

B

C

D

E

**Additional figure 4.** **Uncropped immunoblots of RIG-I blots.** Chemiluminescence channel of C57Bl/6J blot (A), BALB/c blot (C) and C57Bl/6N blot (E). Chemiluminescence and 700 nm channel merge of C57Bl/6J blot (B), BALB/c blot (D) and C57Bl/6N (F), to visualize ladder. Ladder size is indicated.

100 kDa

50 kDa

70 kDa

100 kDa

50 kDa

70 kDa

100 kDa

50 kDa

70 kDa
