# Supplementary material for: C57Bl/6N mice have an attenuated lung inflammatory response to dsRNA compared to C57Bl/6J and BALB/c mice
Source: J Inflamm (Lond). 2023 Feb 21;20:6. doi: 10.1186/s12950-023-00331-4 (PMC9942641; doi:10.1186/s12950-023-00331-4)
Supplement: Supplementary file 5 — Additional file 5: Additional figure 5. Effect of i.n. dsRNA administration on BALF cellular infiltration in mice. dsRNA (100 µg) or saline were i.n. administered to C57Bl/6J, C57Bl/6N or BALB/c mice once per day for three consecutive days, and the total number of lymphocytes (A), neutrophils (B) and macrophages (C) were determined in BALF using cytospin, differential cell counting and cell concentration measurements. Data are presented as mean ± SD. Differences between groups were tested using a 2way ANOVA with Tukey’s multiple comparisons test. P-values of <0.05 are indicted in the graphs, each data point represents one mouse from one experiment (n = 4-5). [file 12950_2023_331_MOESM5_ESM.docx]

**Additional file 5**

**
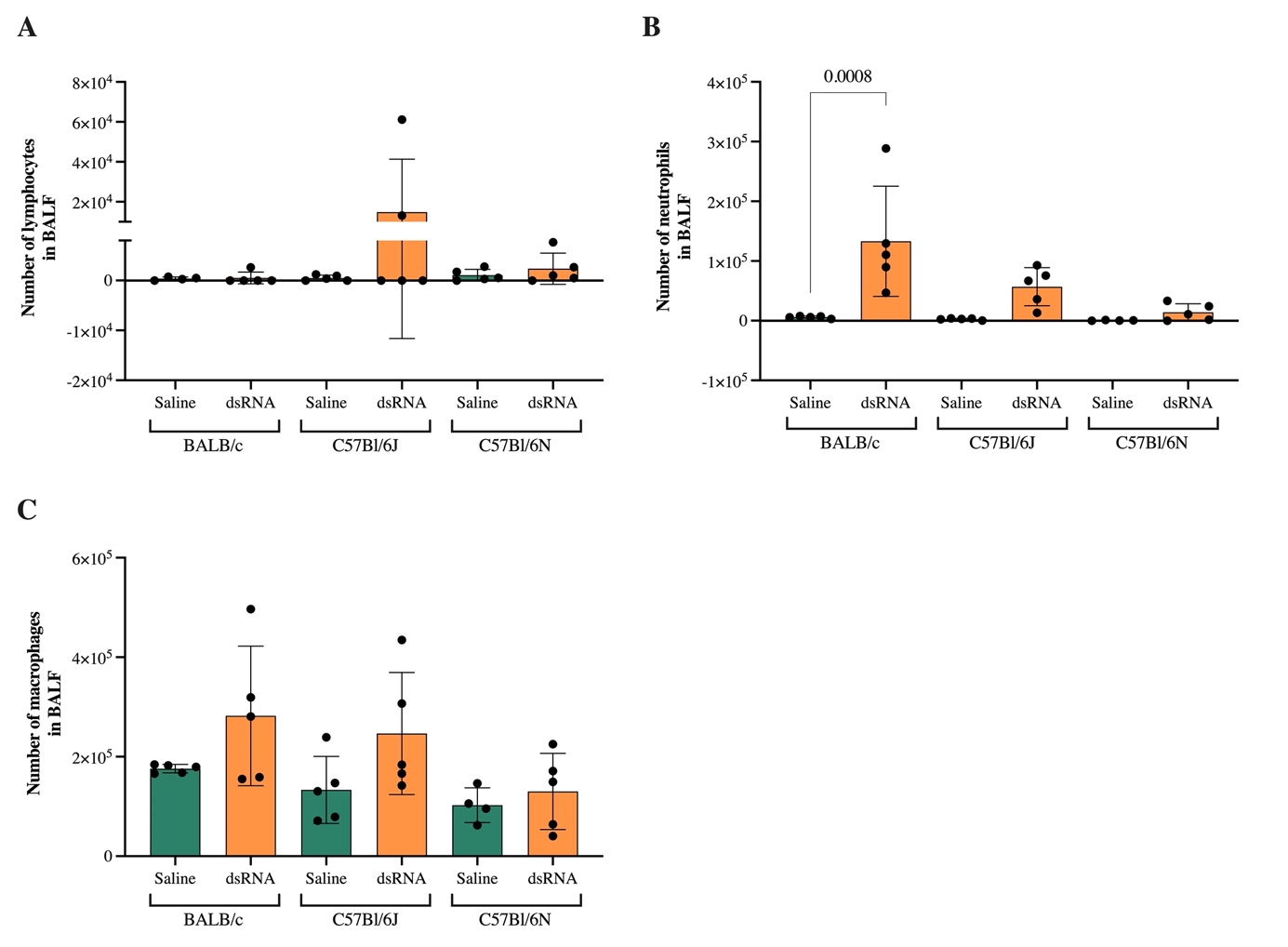
**

**Additional figure 5.** **Effect of i.n. dsRNA administration on BALF cellular infiltration in mice.** dsRNA (100 µg) or saline were i.n. administered to C57Bl/6J, C57Bl/6N or BALB/c mice once per day for three consecutive days, and the total number of lymphocytes (A), neutrophils (B) and macrophages (C) were determined in BALF using cytospin, differential cell counting and cell concentration measurements. Data are presented as mean ± SD. Differences between groups were tested using a 2way ANOVA with Tukey’s multiple comparisons test. P-values of <0.05 are indicted in the graphs, each data point represents one mouse from one experiment (n=4-5).
